# A Novel Setup Technique for Radiation Treatment of a Severely Obese Patient With a Primary Brain Tumor

**DOI:** 10.7759/cureus.42759

**Published:** 2023-07-31

**Authors:** Theodore Arsenault, Kristopher Lyons, Kevin Chaung, Edward Smith, Serah Choi, Gisele Pereira

**Affiliations:** 1 Medical Physics, University Hospitals Cleveland Medical Center, Cleveland, USA; 2 Radiation Oncology, University Hospitals Cleveland Medical Center, Cleveland, USA; 3 Dosimetry, University Hospitals Cleveland Medical Center, Cleveland, USA

**Keywords:** brain tumor, obesity, external beam therapy, radiation therapy, gliosarcoma

## Abstract

Modern external beam radiation therapy (EBRT) techniques rely on the accurate positioning of the patient using the treatment couch. These motorized couches have weight limits that have decreased over time and are not able to support severely obese patients requiring EBRT. We aimed to develop a technique to support obese patients who are above their weight tolerance while accurately delivering radiation treatment to a brain tumor.

This technique was used on a patient receiving adjuvant radiation for gliosarcoma, a variant of glioblastoma. The patient was CT scanned, and the 3D conformal radiation therapy plan was created. A custom treatment couch was created using a transport stretcher, Styrofoam, a CT couch-top, and an IMRT board, which allowed for a thermoplastic mask to be used for a reproducible setup. AP and lateral portal films were taken prior to each treatment to confirm the accuracy of the manual daily setup of the patient on the custom couch.

The patient received 60 Gy in 30 daily fractions of 3DCRT in a reproducible fashion. The average deviation from the isocenter fell within the 10 mm and 8 mm planning margins applied to the clinical target volume (CTV) for the initial and boost fields, respectively. The average daily shifts in the anterior-posterior (AP) direction for the patient were 7.97 (−16.19 to 12.04) mm and 1.98 mm (−1.1 to 4.3) mm for the initial and boost treatments, respectively. The average daily shifts in the superior-inferior (SI) direction were 2.2 (−5.08 to 9.04) mm and 3.88 (−2.9 to 8.0) mm for the initial and boost treatments, respectively.

This novel approach allowed treatment at 60 Gy for a gliosarcoma patient who had previously been denied treatment due to his weight. By utilizing readily available materials within the department, our team was able to create a reproducible setup technique to safely treat the patient.

## Introduction

The prevalence of obesity in the United States was approximately 42% in 2017-2018. Of the top 10 causes of death in the United States in 2009, five were related to obesity, including heart disease, stroke, cancer, diabetes mellitus, and kidney disease [1]. Compared with men and women of average weight, those who were very obese (body mass index [BMI] > 40.0 kg/m^2^) had a relative risk of death from cancer of 1.52 for women and 1.62 for men [2].

Severe obesity presents challenges in simulation, treatment planning, and radiation delivery. As the average weight and BMI of the United States population have increased, commercial linear accelerator couch weight limits have decreased. Potential clinical problems include weight over simulator or treatment couch limits, circumference above CT bore or field of view, poor image quality, achieving an acceptable dose distribution and heterogeneity index given the confines of beam arrangement and body thickness, gantry clearance issues, issues with patient setup and verification, and reliability of external fiducials [3,4]. Treatment couch weight capacity serves as a limiting factor in the treatment of morbidly obese patients, regardless of tumor location. Furthermore, the standard of care radiation dose for glioblastoma is 60 Gy, which exceeds the dose tolerance of several intracranial organs at risk (OARs). In this study, we report a novel setup technique developed in our department to treat a brain tumor patient whose weight exceeded the limit of the treatment couches.

## Technical report

Case presentation

The patient was a severely obese 47-year-old man with a history of stage IV chronic kidney disease who presented with word-finding difficulty, short-term memory loss, and seizures. CT scans showed a 5.5-cm mass in the left temporal lobe with surrounding vasogenic edema and an 8-mm midline shift. His body habitus initially precluded getting a brain MRI. He underwent a left temporal craniotomy for gross tumor resection. Pathology revealed gliosarcoma (a rare variant of glioblastoma), WHO grade IV, negative for O^6^-methylguanine-DNA methyltransferase (MGMT) promoter methylation. Postoperative CT showed postsurgical changes with the improvement of the midline shift to 4 mm. Initially, he was unable to undergo postoperative MRI as an inpatient because of his body habitus and could not receive intravenous contrast due to acute chronic kidney disease. The patient recovered well and was referred to radiation oncology for adjuvant therapy. His case was discussed at a multidisciplinary neuro-oncology tumor board, and standard adjuvant therapy with concurrent chemoradiation to 60 Gy in 30 fractions was recommended because of his young age, performance status (KPS 60), and desire to seek aggressive therapy despite his severe obesity. He was referred to our department as there were no centers in the region that could accommodate treatment for his weight. The patient’s BMI was 63.6 kg/m^2^, he stood 6’3" tall, and he weighed 231.3 kg (510 pounds), which exceeded our radiation treatment couch’s weight tolerance of 200 kg (440 pounds).

Simulation

The patient was simulated in a head-first supine position with a thermoplastic mask pinned to an intensity-modulated radiation treatment (IMRT) board on the simulation couch. Our CT simulator is a Phillips Brilliance Big Bore, which is a 16-slice helical scanner with a "large bore" 80 cm gantry opening that has been fitted with a reinforced carbon fiber couch top with a weight limit of 295 kg. A treatment planning MRI of the brain was done using a Siemens Espree 1.5T MRI system with a 70 cm bore and a 250 kg weight tolerance. The patient’s abdominal girth was a limiting factor in the choice of MR scanner size.

Treatment planning

**Figure 1:**
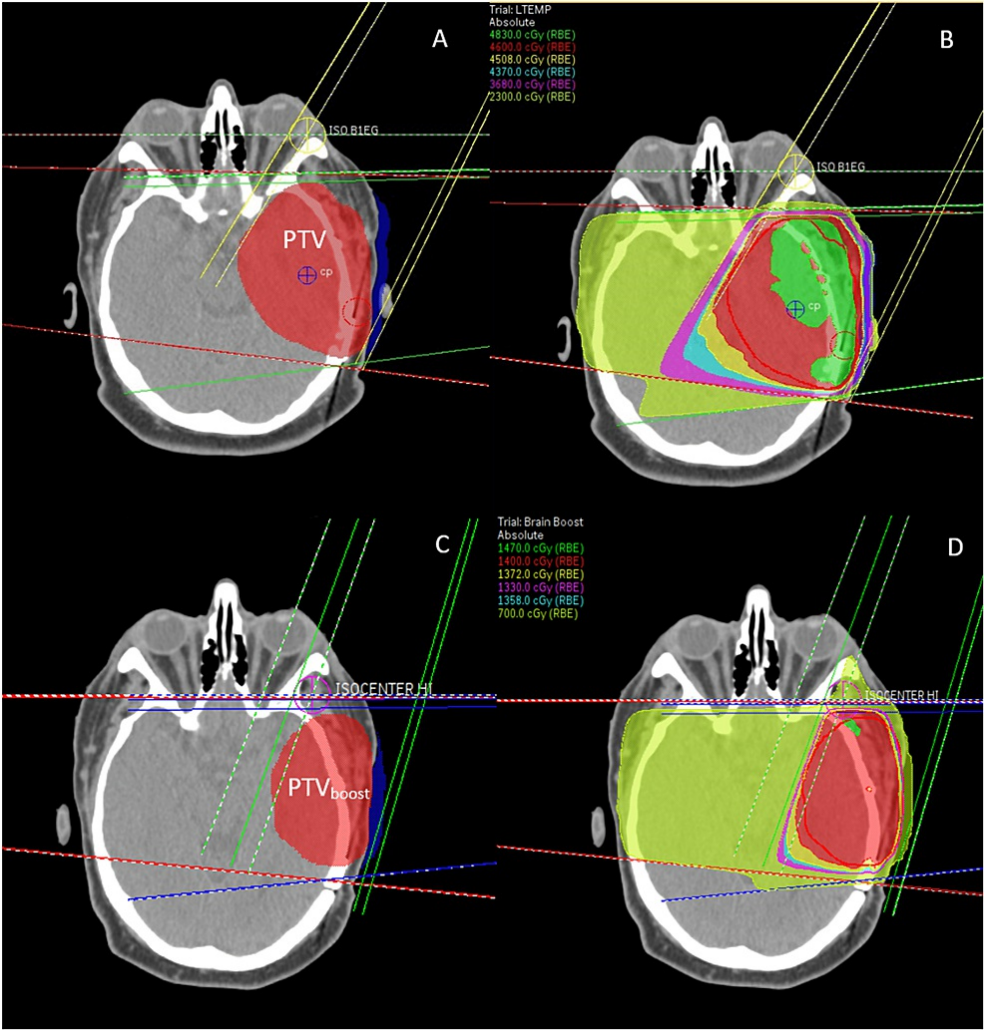
Opposed lateral fields matched behind the optics and an oblique field shown in (A) and (C) for the initial and boost plans respectively. The dose distributions for the initial and boost plans are shown in (B) and (D), respectively.

Although volumetric modulated arc therapy (VMAT) is the preferred treatment method for gliosarcoma patients receiving 60 Gy, treatment on a custom stretcher limits the available angles for treatment due to collisions. Therefore, three-dimensional conformal radiation therapy was implemented. A three-field technique planned in Pinnacle version 16.2 (Figure 1) was used to treat the target to 46 Gy at 2 Gy per fraction, followed by a cone-down boost for a total dose of 60 Gy at 2 Gy per fraction on an Elekta Synergy equipped with an agility head.

The right lateral field used a 15 MV beam, while the left anterior oblique and left lateral fields used 6 MV beams to minimize the dose to normal tissue in the right hemisphere. Posterior beams were not possible because of collision issues between the accelerator head and the custom couch. It is routine to also treat with different couch kicks in order to spare healthy tissue. However, this could not be done due to limitations on acquiring accurate daily images for clinical setup. Bolus was placed on the left side of the patient’s head to bring the dose laterally towards the surface for a gliosarcoma. A planning target volume (PTV) margin of 10 mm and 8 mm was set around the clinical target volume (CTV) for the initial and boost plans, respectively, based on guidance from physics on the best estimate of setup uncertainty. The PTV margin was reduced to 8 mm for the boost due to several factors, including setup improvement with the introduction of an IMRT headboard to the custom couch and the familiarity of the therapists with the daily setup technique.

Treatment delivery

**Figure 2:**
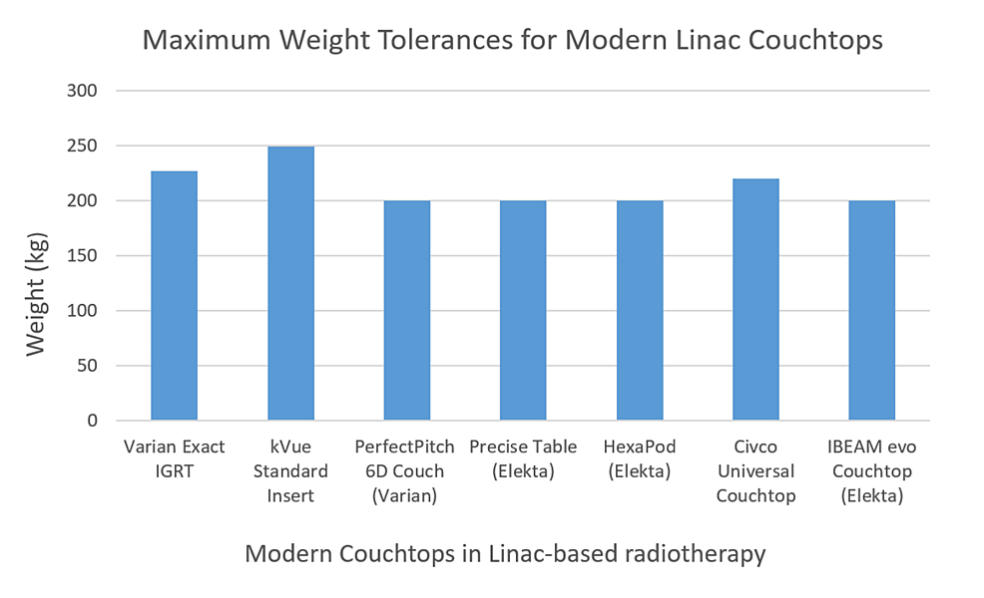
Comparison of LINAC couch tops from various vendors. As couch top complexity evolves, i.e., more degrees of freedom, the weight limit decreases.

At our center, the couch weight limitation is 200 kg (440 lbs) evenly distributed, which is similar to other vendors (Figure 2) [5-9].

**Figure 3:**
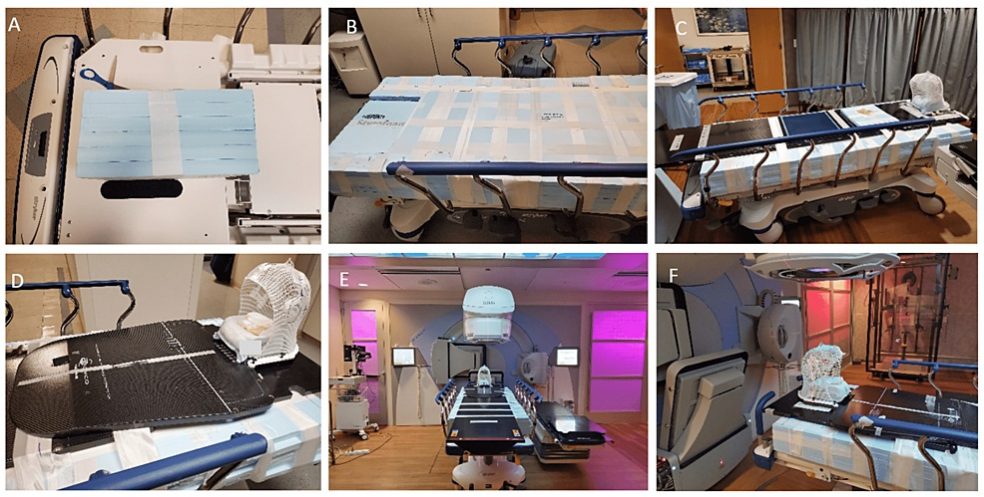
Construction of custom bed. Progression of building the customized patient bed from A through F. The bottom row includes the installation of the IMRT board used to properly fasten the patient’s thermoplastic mask to the treatment couch.

A specially modified Stryker Prime series transport stretcher (306 kg weight limit) was used in place of the treatment couch to accommodate the patient during treatment. The cushion was removed from the stretcher to reduce setup uncertainty from uneven weight distribution. In place of the cushion, a series of 30 cm × 30 cm × 2.5 cm Styrofoam blocks were fastened together to create a Styrofoam bed 15 cm in height. The height of the Styrofoam bed was chosen to provide excess room to raise and/or lower the patient into the treatment position, as the Stryker stretcher alone did not provide enough lift to align the patient to the isocenter. An unused CT couch top was fastened on top of the Styrofoam couch. When planning the boost field, we pinned an IMRT board to the CT couch-top and created a new thermoplastic mask to create a more reproducible day-to-day setup (Figure 3).

Verification

**Figure 4:**
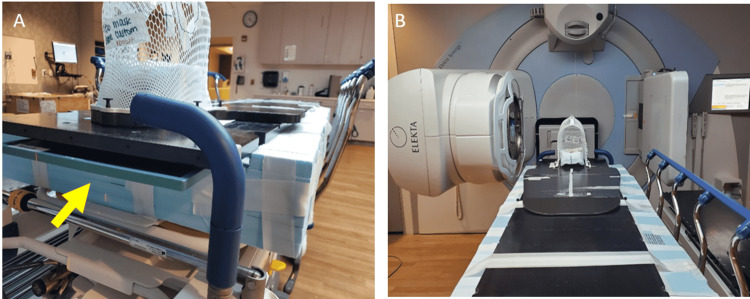
Setup verification. Depiction of patient setup for the AP and lateral portal images. The yellow arrow (A) shows the portable Fujifilm CR detector in position for AP portal images. A slot was carved out of the Styrofoam section of the couch for the CR detector to be placed; (B) the ability to take a lateral part film with the LINAC's onboard imaging.

The patient was initially localized using room lasers, and then daily anterior-posterior (AP) and lateral port films were used for position verification. Because of the patient setup, lateral films were taken using Elekta’s onboard iView imaging system. The MV panel could not extend underneath the couch with the patient in position, so a slot was carved out of the superior side of the Styrofoam couch where a Fujifilm cassette could be placed to image the AP port (Figure 4). The physician and physicist reviewed and approved the images prior to the delivery of each fraction.

Manual manipulation of the couch using the Stryker stretcher’s side control hydraulic pumps for the AP direction and translational motion of the Stryker stretcher in the SI and lateral directions were used to reposition the patient based on portal imaging.

**Figure 5:**
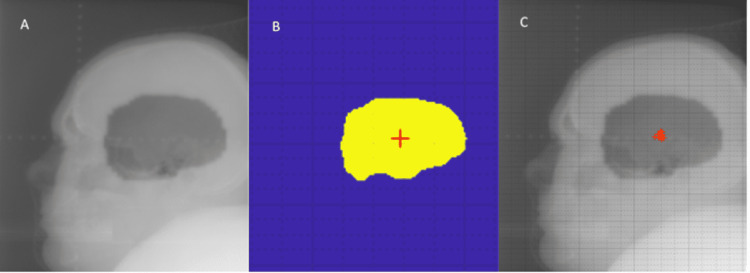
Retrospective analysis of patient setup. Image mask used in a retrospective review of patient setup. (A) A lateral film taken for treatment setup using the onboard imaging system. Centroids shown in (B) and (C) of the mask were compared against the planned isocenter as a reference to find daily deviations from the plan; (C) the clustering of image mask centroids in relation to each other verifying positioning accuracy.

A retrospective review of the lateral films using an in-house MATLAB 2019A program determined the average shifts in the anterior-posterior direction and in the superior-inferior (SI) direction, as shown in Table 1. The program created a mask of the field shape from the portal image that had been rigidly registered to the digitally reconstructed radiography (DRR) from the treatment planning system (Figure 5).

**Table 1:** Mean deviations and ranges of mask centroids from planned isocenter in mm in the anterior-posterior and superior-inferior directions. AP: anterior-posterior, SI: superior-inferior.

	AP	SI
Left lateral (mm)	7.2 (−1.9 to 12.0)	1.9 (−5.1 to 8.0)
Right lateral (mm)	8.8 (−16.2 to 2.6)	2.5 (−4.5 to 9.4)
Left lateral boost (mm)	2.0 (−1.1 to 4.3)	1.5 (−2.9 to 6.9)
Right lateral boost (mm)	1.9 (1.2 to 2.8)	6.3 (2.1 to 8.0)

Results

The Styrofoam couch maintained both its shape and thickness; weekly measurements of the Styrofoam compression showed a less than 1 mm change over the entirety of the treatments. Table 1 shows the results of the daily setup variation between the AP and lateral images.

## Discussion

Our study introduced a new method to aid obese patients who surpass the weight capacity of typical treatment couches during external beam radiation therapy (EBRT). The approach successfully delivered accurate radiation treatment to a severely obese patient with gliosarcoma who had previously been denied treatment due to his weight.

The prevalence of obesity in the United States has been steadily increasing, posing significant challenges in various aspects of radiation therapy, including simulation, treatment planning, and delivery [1,2]. Commercial linear accelerator couches have been designed with decreasing weight limits, further complicating the treatment of morbidly obese patients. Our patient presented with a BMI of 63.6 kg/m² and weighed 231.3 kg, exceeding the weight tolerance of our treatment couch. This excess necessitated the development of an alternative setup technique.

Utilization of the specially modified Stryker Prime series transport stretcher to accommodate the patient replaced the need for a conventional treatment couch. The proposed technique builds upon past work in treating severely obese patients by adding additional motion management and the availability to take multiple images during treatment setup [3]. Onboard imaging allowed for accurate patient positioning before treatment and provided a retrospective analysis of the setup to improve the setup technique. Daily AP and lateral portal films were taken before treatment to verify the patient's position. Due to the limitations of the Styrofoam couch, the AP films were captured by carving out a slot in the couch to accommodate a Fujifilm cassette. The physician and physicist reviewed and approved each set of images before treatment delivery. Based on portal imaging feedback, therapists made manual adjustments to the couch using the Stryker stretcher's side control hydraulic pumps. During the course of treatment, the patient was closely monitored using the treatment vault’s audio-visual system, and intra-fraction imaging was performed when any movement was detected.

Our retrospective analysis of the port films using an in-house MATLAB program revealed the average daily shifts in the AP and SI directions (Figure 5). The patient demonstrated acceptable deviations from the planned isocenter, with the average daily shifts falling within the planning margins for both the initial and boost treatments. The AP shifts ranged from −16.19 to 12.04 mm for the initial treatment and −1.1 to 4.3 mm for the boost treatment, while the SI shifts ranged from −5.08 to 9.04 mm for the initial treatment and −2.9 to 8.0 mm for the boost treatment (Table 1). Variability in the AP direction was likely more significant than in the SI direction because the Stryker stretcher's side control hydraulic pump mechanism was more challenging to manipulate. As treatment progressed, confidence in the treatment setup grew with the familiarity of the patient setup.

Our novel setup technique allowed us to deliver the prescribed dose of 60 Gy in 30 daily fractions to the gliosarcoma patient, despite their weight exceeding the treatment couch's weight limit. We achieved a reproducible setup that ensured accurate treatment delivery by utilizing readily available materials within our department. The success of this approach highlights the importance of adapting and innovating in response to the challenges posed by obesity in radiation therapy.

While our technique proved effective, several limitations should be acknowledged. First, a 3D conformal radiation therapy technique was necessary instead of the preferred VMAT method due to collision issues with the custom couch. This limited the available treatment angles and potentially affected dose distribution. Our group considered IMRT; however, after consultation with our institution's physician and physics team, our group decided that a 3D conformal radiation plan would be the most conservative, given that this was the first time performing this procedure at our center. Static intensity-modulated radiation therapy may be considered for future patients if deemed acceptable. Second, the absence of different couch kicks restricted our ability to spare healthy tissue. During treatment, the patient developed left-sided tinnitus and skin erythema, which was expected due to the target location and inability to spare a high dose to the left cochlea. Additionally, the daily setup verification relied on manual adjustments based on portal imaging, which may have introduced uncertainties. Our group has provided a means to retrospectively review random setup errors by analyzing port films. It is recommended to assess the construction of the custom-made couch and review localization images as mentioned above to minimize any localization errors.

## Conclusions

Our novel technique significantly advances radiation treatment for severely obese patients, addressing a critical issue presented to medical centers. Using inexpensive and readily available materials to construct the treatment bed allows for easy replication for Cancer Centers, enhancing access to accurate radiation therapy for this underserved patient population. Additionally, the retrospective assessment technique provides a valuable means to refine and improve the treatment process for future patients.

The success of this approach highlights the importance of innovative solutions to overcome the challenges presented by severe obesity in radiation therapy. By adapting existing resources and materials, we have successfully provided appropriate treatment to previously excluded patients, ensuring equitable access to effective cancer care for all individuals, regardless of their body habitus. Further studies and experiences with similar setups will strengthen the validity and broaden the applicability of this technique, promoting better outcomes, better organ-at-risk sparing, and enhanced care for severely obese patients undergoing radiation therapy.
